# ELDER ABUSE AND NEGLECT IN URBAN AND RURAL AREAS IN CHINA: PREVENTION STRATEGIES

**DOI:** 10.1093/geroni/igz038.3428

**Published:** 2019-11-08

**Authors:** Chen Chen, Liu Yue, Hong Mi

**Affiliations:** 1 Institute for Population and Development Studies, Zhejiang University, Hangzhou, Zhejiang, China; 2 Institute for Population and Development Studies, Hangzhou, Zhejiang Province, China; 3 School of Public Affairs, Zhejiang University, Hangzhou, Zhejiang Province, China

## Abstract

With acceleration of the ageing population globally, more and more governments are concerned about the potential increase in elder abuse and neglect (EA/N). Recently the National Office for Ageing and the provincial offices for ageing conducted a survey of 224,352 Chinese over the age of 60 years using household interviews to assess economy, health, service, social participation, culture, rights protection, livable environment, etc. Author’s analysis of this data shows that 54% of the elderly people interviewed experienced physical and mental abuse or intimidation, and 6.95% of them felt that their legal rights were violated. Data also supports that the occurrence of EA/N was significantly correlated to self-care ability, economic status, and urban and rural regions of the elders. The researcher will discuss the practice and policy implications for the prevention of EA/N.

